# Astragaloside IV ameliorates peritoneal fibrosis by promoting PGC‐1α to reduce apoptosis in vitro and in vivo

**DOI:** 10.1111/jcmm.17871

**Published:** 2023-07-26

**Authors:** Mingxia Xie, Bohou Xia, Lan Xiao, Dun Yang, Zhenghong Li, Hanqing Wang, Xiaoye Wang, Xi Zhang, Qinghua Peng

**Affiliations:** ^1^ College of Clinical Medicine Hunan University of Chinese Medicine Changsha People's Republic of China; ^2^ College of Traditional Chinese Medicine Hunan University of Chinese Medicine Changsha People's Republic of China; ^3^ College of Pharmacy Hunan University of Chinese Medicine Changsha People's Republic of China; ^4^ Departments of Nephrology, Jiangsu Province Hospital of Chinese Medicine Affiliated Hospital of Nanjing University of Chinese Medicine Nanjing People's Republic of China; ^5^ College of Pharmacy Ningxia Medical University Yinchuan People's Republic of China

**Keywords:** apoptosis, Astragaloside IV, mitochondria, peritoneal fibrosis, PGC‐1α

## Abstract

Prolonged exposure of the peritoneum to high glucose dialysate leads to the development of peritoneal fibrosis (PF), and apoptosis of peritoneal mesothelial cells (PMCs) is a major cause of PF. The aim of this study is to investigate whether Astragaloside IV could protect PMCs from apoptosis and alleviate PF. PMCs and rats PF models were induced by high glucose peritoneal fluid. We examined the pathology of rat peritoneal tissue by HE staining, the thickness of rat peritoneal tissue by Masson's staining, the number of mitochondria and oxidative stress levels in peritoneal tissue by JC‐1 and DHE fluorescence staining, and mitochondria‐related proteins and apoptosis‐related proteins such as PGC‐1α, NRF1, TFAM, Caspase3, Bcl2 smad2 were measured. We used hoechst staining and flow cytometry to assess the apoptotic rate of PMCs in the PF model, and further validated the observed changes in the expressions of PGC‐1α, NRF1, TFAM, Caspase3, Bcl2 smad2 in PMCs. We further incubated PMCs with MG‐132 (proteasome inhibitor) and Cyclohexylamine (protein synthesis inhibitor). The results demonstrated that Astragaloside IV increased the expression of PGC‐1α by reducing the ubiquitination of PGC‐1α. It was further found that the protective effects of Astragaloside IV on PMCs were blocked when PGC‐1α was inhibited. In conclusion, Astragaloside IV effectively alleviated PF both in vitro and in vivo, possibly by promoting PGC‐1α to enhance mitochondrial synthesis to reduce apoptotic effects.

## 1 INTRODUCTION

Chronic kidney disease has become one of the global public health problems, with a high prevalence of end‐stage renal disease reported in countries such as the United States, Japan and China.^1^ Peritoneal dialysis (PD) is recognized and widely used as a therapy treatment for end‐stage renal disease that utilizes the peritoneum as a physical barrier for the exchange of toxic substances and fluids. During PD treatment, the biocompatibility of the dialysate initiates the activation of peritoneal intrinsic cells. Over time, the loss of function of peritoneal mesothelial cells (PMCs) may lead to disturbances in peritoneal integrity and homeostasis, thereby promoting the progression of peritoneal fibrosis (PF) and ultimately leading to failure of PD treatment.^2^, ^3^, ^4^ How to alleviate PF, improve the effectiveness of peritoneal therapy and improve the quality of dialysis is currently an important issue in the nephrology community. Therefore, prevention of PF in patients with PD is one of the priorities of PD treatment and research.^3^

Current research suggests that damage to PMCs may be caused by two factors: one is the inhibition of the growth and proliferation of PMCs, and the other is the apoptosis and necrosis of PMCs, in which the apoptosis of PMCs is particularly important. Apoptosis is a direct factor in PF, while mitochondrial dysfunction leads to abnormal redox signalling, which in turn leads to apoptosis. It was found that peroxisome proliferator‐activated receptor gamma co‐activator (PGC‐1α), an important intracellular nuclear receptor transcriptional co‐activator, is a major regulator of intracellular mitochondrial biosynthesis and oxidative metabolism. PGC‐1α can regulate mitochondrial related proteins synthesis including respiratory chain complex, through the activation of nuclear respiratory factor 1 (NRF1)^5^; additionally, it also regulates mitochondrial DNA replication and transcription by binding to the TFAM promoter. Therefore, exploring the pathways by which PGC‐1α regulates mitochondrial influences on apoptosis may be a novel approach for the prevention and treatment of PF.

Astragaloside IV is a natural clover glycosides derived from the traditional Chinese herb *Radix Astragali* root of *Astragalus membranaceus* (Fisch.) Bge. Var. *mongholicus* (Bge.) Hsiao. *Radix Astragali* is the widely used traditional Chinese medicine for the treatment of renal disease and diabetes according to activate blood and resolve stasis the theory of traditional Chinese medicine (TCM).^6^, ^7^ Several comparative studies have confirmed the pleiotropic function of Astragaloside IV in the progression of various pathologies. Wang et al. found that Astragaloside IV antagonizes M2 phenotype macrophage polarization‐evoked ovarian cancer cell malignant progression by suppressing the HMGB1‐TLR4 axis.^8^ It is also used to prevent and treat cardiac injury.^9^, ^10^ Recent studies show that Astragaloside IV is used to treat pulmonary fibrosis and renal fibrosis.^11^, ^12^ Among them, the anti‐fibrotic mechanism of Astragaloside IV is mainly manifested in the inhibition of the TGF‐β/Smads pathway.^13^

In our previous study, we found that Astragaloside IV affects PF through other pathways besides the TGF‐β/Smads pathway, and that Astragalus inhibits apoptosis and scavenges oxygen radicals in PMCs.^14^ We therefore speculate that Astragaloside IV is likely to also inhibit PF via the mitochondrial biosynthesis‐ROS‐apoptosis‐related pathway in PMCs. In the present study, we tested the hypothesis by inducing fibrosis in rats and PMCs with high glucose and then analysing the inhibitory effects of Astragaloside IV on the fibrous transformation of PMCs.

## 2 MATERIALS AND METHODS

### 2.1 Material

**FIGURE 1:**
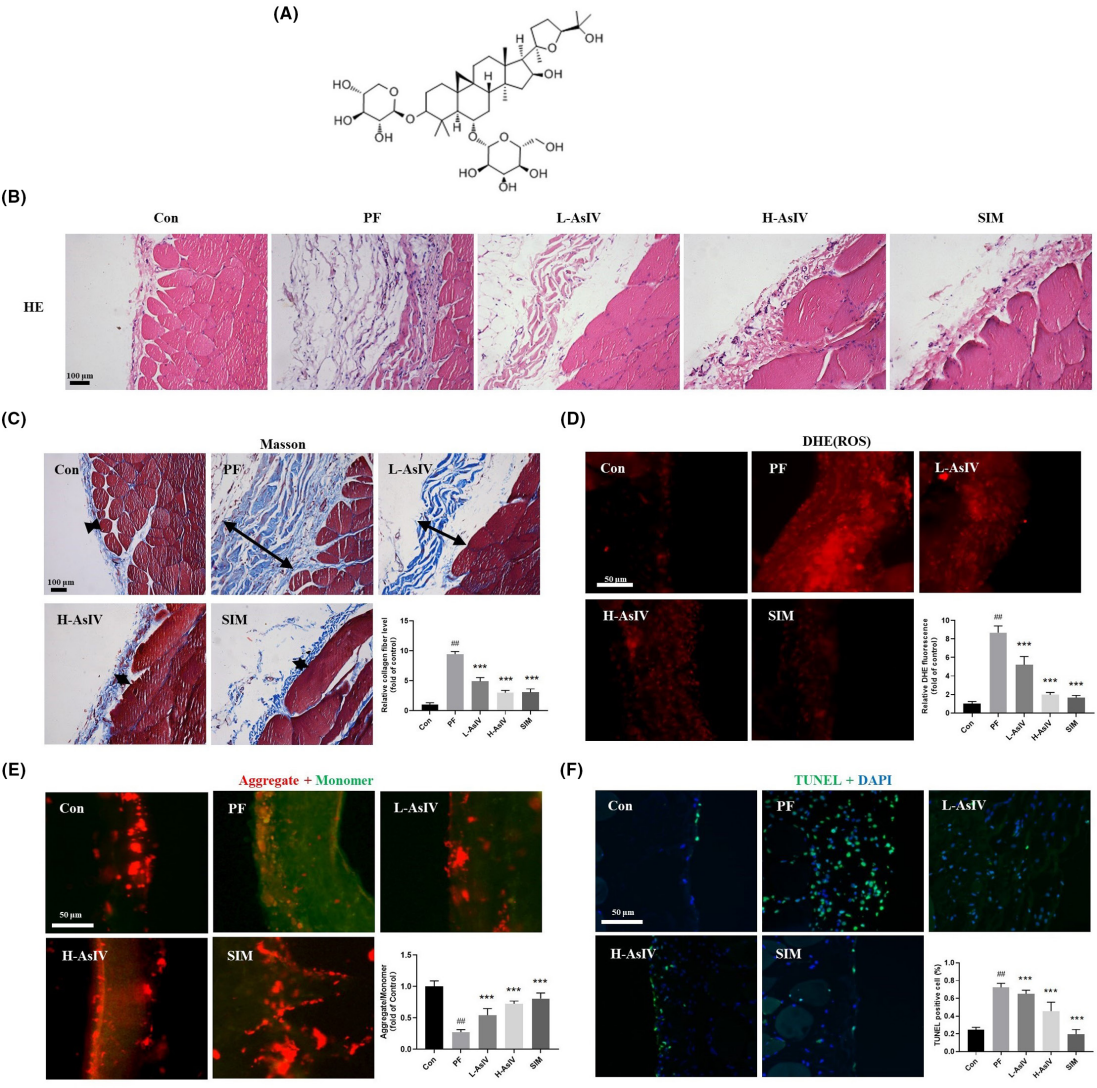
Astragaloside IV reduces peritoneal tissue thickness in rats with peritoneal fibrosis. (A) The chemical structure of Astragaloside IV. (B) Representative microscopic images of haematoxylin–eosin staining of peritoneal tissues (*n* = 3). (C) Representative microscopic images of Masson's staining of peritoneal tissues (*n* = 3). (D) Representative fluorescent images of peritoneal tissue stained with DHE (ROS) (*n* = 3). (E) Representative fluorescent images of JC‐1 staining of peritoneal tissue, red for aggregate and green for monomer, the relative ratio of red fluorescence/green fluorescence is a measure of mitochondrial membrane potential (*n* = 3). (F) Apoptosis detection in peritoneal tissue (*n* = 3). Con: Control group; PF: PF model group; L‐AsIV: 20 mg/kg/day Astragaloside IV group; H‐AsIV: 40 mg/kg/day Astragaloside IV group; SIM: 20 mg/kg/day Simvastatin group. Results are representative of three independent experiments values are expressed as mean ± SD. ^#^ *p* < 0.05 and ^##^ *p* < 0.01 versus Control group; *p < 0.05 and **p < 0.01 versus PF group.

Astragaloside IV (C_41_H_68_O_14_, A800922, purity ≥98.5%) was purchased from Maclin Inc. and the chemical structure is shown as Figure 1A. ROS fluorescent probe‐DHE (R001, Vigorous Biotechnology) used for detection of ROS in tissues and DCFH‐DA (KM0062) used for detection of cellular ROS was purchased from Beijing Biolab Technology Co. The antibodies used in this study including NRF1 (ab175932, abcam), PGC‐1α (ab106814, abcam), TFAM (ab252432, abcam), Caspase3 (9662, CST), cleaved‐caspase3 (ab32042, abcam), Bax (ab111391, abcam), Bcl2 (2872, CST), α‐SMA (A5228, sigma), p‐smad2 (ab280888, abcam), smad2 (ab40855, abcam), MT‐CO1 (ab203912, abcam), MT‐ND6 (PA5‐109993, Invitrogen) and MT‐ATP6 (PA5‐116789, Invitrogen). MG‐132 (M7749) and Cycloheximide (C7698) were purchased from Sigma.

### 2.2 Rats and experimental protocol

Female Sprague–Dawley rats purchased from ALF Biotechnology Co. Ltd weighed 220–260 g were housed at 21–23°C with a 12 h light/dark cycle. All animal experiments were approved by the Animal Ethics Committee of Hunan University of Chinese Medical Ethical Clearance Certificate (No. 2021‐15, 20 September 2021). Rats were randomly separated into five groups (*n* = 9 in each group): Con group (normal rats without treatment, control group), PF group (negative group, PF model rats without treatment), low concentration of Astragaloside IV group (L‐AsIV, PF model rats received 20 mg/kg/day Astragaloside IV), high concentration of Astragaloside IV group (H‐AsIV group, PF model rats received 40 mg/kg/day Astragaloside IV) and SIM group (positive control, PF model rats received 20 mg/kg/day Simvastatin). Simvastatin could protect PD rats from PF by inhibiting tissue factor expression, and it was used as a positive control in the present study.

The cause of PF model was simulated by intraperitoneal injection of 4.25% glucose peritoneal permeate into rats, and all rats except control rats were injected with saline.^15^ This modelling procedure of 4.25% glucose peritoneal permeate injection lasted 34 days and Astragaloside IV and Simvastatin were administered intraperitoneally to the experimental groups from Day 15. On Day 35, the animals were sacrificed by injection of 200 mg/kg of pentobarbital sodium and peritoneal tissues were retained for pathological examination and peritoneal proteins were routinely extracted for Western blot analysis. The study guideline of animal experiments was confirmed by the Institutional Animal Care and Use Committee of Hunan University of Chinese Medicine.

### 2.3 Haematoxylin–eosin (HE) and Masson's staining

The peritoneal tissues from rats were washed in saline, fixed in formalin, dehydrated transparently and then embedded in paraffin. Sections were attached to thin slides for drying, stained with haematoxylin for 5 min and discoloured in hydrochloric acid ethanol, followed by eosin re‐staining for 2 min. The transparent sealed sections were placed under a microscope to observe the pathological changes.

Masson's staining is one of the staining methods used to show fibre in tissues. First, the dehydrated peritoneal tissue sample was stained with haematoxylin for 5 min, fractionated with 1% hydrochloric acid for a few seconds and rinsed with running water. The samples were then treated with ponceau in aqueous solution for 5 min and then re‐stained with aniline blue for 5 min, differentiated with 1% of glacial acetic acid for 1 min, then dehydrated and sealed under the microscope.

### 2.4 Detection of ROS

Dihydroethidium (DHE) was used to assess the production of superoxide anion in tissue cells. Briefly, treated cells were washed and incubated with 5 μM of DHE for 30 min at 37°C protected from light. The cells were then subjected to fluorescence microscopy.

Levels of intracellular ROS were detected by DCFH‐DA. Treated PMCs were incubated in 1640 medium containing DCFH‐DA (10 μM) for 30 min. After removing DCFH‐DA from the culture medium, photographs were taken under a fluorescent microscope, and the relative DCF fluorescence intensity of the cells was analysed. Data from three independent experiments were quantified.

### 2.5 Detection of apoptosis

Apoptosis is generally defined as a programmed cell death that occurs when cells are regulated. Apoptosis is manifested in the early stages as ectopic cell membrane phosphatidylserine and reduction of mitochondrial membrane potential, and in the later stages as nuclear condensation and rupture, which can be detected by Annexin V, JC‐1 and TUNEL assays, respectively.

Apoptosis was detected using the Annexin V‐FITC Apoptosis Detection Kit. Briefly, cells collected were digested with tryptic digest, washed twice with pre‐cooled PBS and then reacted with Annexin V‐FITC and propidium iodide (PI) for 15 min at room temperature in dark. The stained cells were subjected to flow cytometry.

In normal mitochondria, JC‐1 aggregates in the mitochondrial matrix to form a polymer which emits intense red fluorescence, whereas in apoptotic cells, the mitochondrial transmembrane potential is depolarized and JC‐1 is released from the mitochondria in reduced concentration, reversing to a monomeric form that emits green fluorescence. The treated cells were collected, to which JC‐1 was added, and incubated for 20 min at 37°C. The supernatant was removed by centrifugation and cells were resuspended in PBS and observed under a fluorescent microscope.

### 2.6 Cells culture and treatment

Rat PMCs (purchased from Procell Life Science & Technology Co., Ltd.) were cultured in RPMI 1640 medium containing fetal bovine serum (FBS) with 5% CO_2_ at 37°C. PMCs belong to a single layer of flattened epithelium in the epithelial tissue, a layer of cells covering the surface of the peritoneum. Cells were grouped and after 24 h of normal culture, the serum‐free RPMI 1640 medium was replaced for 24 h to synchronize the cell cycle. Administration groups were given different concentrations of Astragaloside IV pre‐administration for 2 h, followed by co‐culture with the modelling agent 4.25% glucose peritoneal solution for 24 h. The specific arrangements for each group were as follows: Con group (control group, normal culture medium), PF group (PF model group, 4.25% glucose peritoneal fluid: RPMI 1640 medium = 1:1 (v:v)), 10‐ AsIV group (4.25% glucose peritoneal fluid: RPMI 1640 medium = 1:1 (v:v), plus 10 μg AsIV), 20‐ AsIV group (4.25% glucose peritoneal fluid: RPMI 1640 medium = 1:1 (v:v), plus 20 μg AsIV), 30‐ AsIV group (4.25% glucose peritoneal fluid: RPMI 1640 medium = 1:1 (v:v), plus 30 μg AsIV).

### 2.7 Western blot analysis

Proteins from tissues and cells were extracted using lysis buffer and proteins were quantified by Bradford method. Equal amounts of proteins were loaded and separated by electrophoresis in 10% SDS‐PAGE gel and transferred to polyethylene difluoride membranes. The membranes were incubated with primary antibody overnight at 4°C first, and then incubated with corresponding secondary antibody for 2 h. The blots were then developed by ECL chemiluminescence assay kit (SW2020, Solarbio) and analysed using ImageJ software.

### 2.8 Marking mitochondria

Mito‐Tracker Green (C1048) brought from Beyotime is a mitochondrial green fluorescent probe that can be used for mitochondria‐specific fluorescent staining of living cells. In contrast to Rhodamine 123 or JC‐1, Mito‐Tracker Green does not depend on the mitochondrial membrane potential for mitochondrial staining. Briefly, the treated cells were collected, the cell culture medium was removed and then incubated with prepared Mito‐Tracker GREEN working solution (anhydrous DMSO diluted to 1 mM) for 25 min at 37°C. The Mito‐Tracker Green staining working solution was removed and fresh cell culture pre‐warmed at 37°C was added. Mitochondria were observed to be brightly and strongly fluorescently stained by fluorescence microscopy.

The relative values of the control groups were observed by flow cytometry at a wavelength of 520 mm in each group.

### 2.9 Co‐Immunoprecipitation (Co‐IP) assay

Collected cells were lyzed and centrifuged for immunoprecipitation. The supernatant was incubated overnight with anti‐PGC‐1 antibody and then incubated for 1 h with Pierce™ Protein A/G Agarose Magnetic Beads (78609, Thermo). The immune complexes were washed three times with lysis buffer and boiled in buffer for 5 min before being subjected to Western blot detection.

### 2.10 siRNA transfection assay

To further confirm the role of PGC‐1α on the regulation of Astragaloside IV on PF, PGC‐1α siRNA was used in the study, and the sequence of siRNA‐PGC‐1α was shown as follows: sense: 5′‐GGCACGCAAUCCUAUUCAUTT‐3′; and antisense: 5′‐AUGAAUAGGAUUGCGUGCCTT‐3′. Dilute 20 pmol siRNA and 1 μL LipoGene™ 2000 PLus Transfection Reagent (L7003, Us Everbright Inc.) separately with 50 μL FBS‐free medium and mix them to obtain transfection solution. Cells were transfected with control siRNA and PGC‐1α siRNA, respectively, and then transfection solution was added and gently shaken. The status of the cells was observed by fluorescence microscopy half a day after transfection assay, and the expression of PGC‐1α in each group was measured after 48 h of culture.

### 2.11 Statistical analysis

Data were presented as mean ± SD. All experimental data were analysed using Graph Prism 8.02. Statistical differences were determined by one‐way analysis of variance followed by Dunnett multiple comparisons test. *p* < 0.05 was considered statistically significant.

## 3 RESULTS

### 3.1 Astragaloside IV reduces the thickness of peritoneal tissue in PF rats

The peritoneal tissue in the control rats consisted of a single layer of peritoneum and thin connective tissue, whereas the peritoneal tissue in the PF group showed a significantly thickened, while the peritoneal thickness of Astragaloside IV group was significantly lower than that of the PF group, and the peritoneal thickness of the H‐As‐IV group was similar to that of the positive drug SIM group (*p* < 0.05, Figure 1B,C). As shown in Figure 1D,E, the increase of tissue thickness of the PF group was accompanied by the increased level of ROS and decreased potential of mitochondrial membrane, both of which are signs of pre‐apoptosis. Further Tunel assay demonstrated this, with lower apoptosis rates in the Astragaloside IV treatment groups than that in the PF group (Figure 1F). Astragaloside IV treatment not only reduced peritoneal thickness, but also decreased the accumulation of ROS and apoptosis, increased potential of mitochondrial membrane.

### 3.2 Astragaloside IV changes relative expressions of mitochondrial synthesis, apoptosis and PF‐associated proteins in rats

**FIGURE 2:**
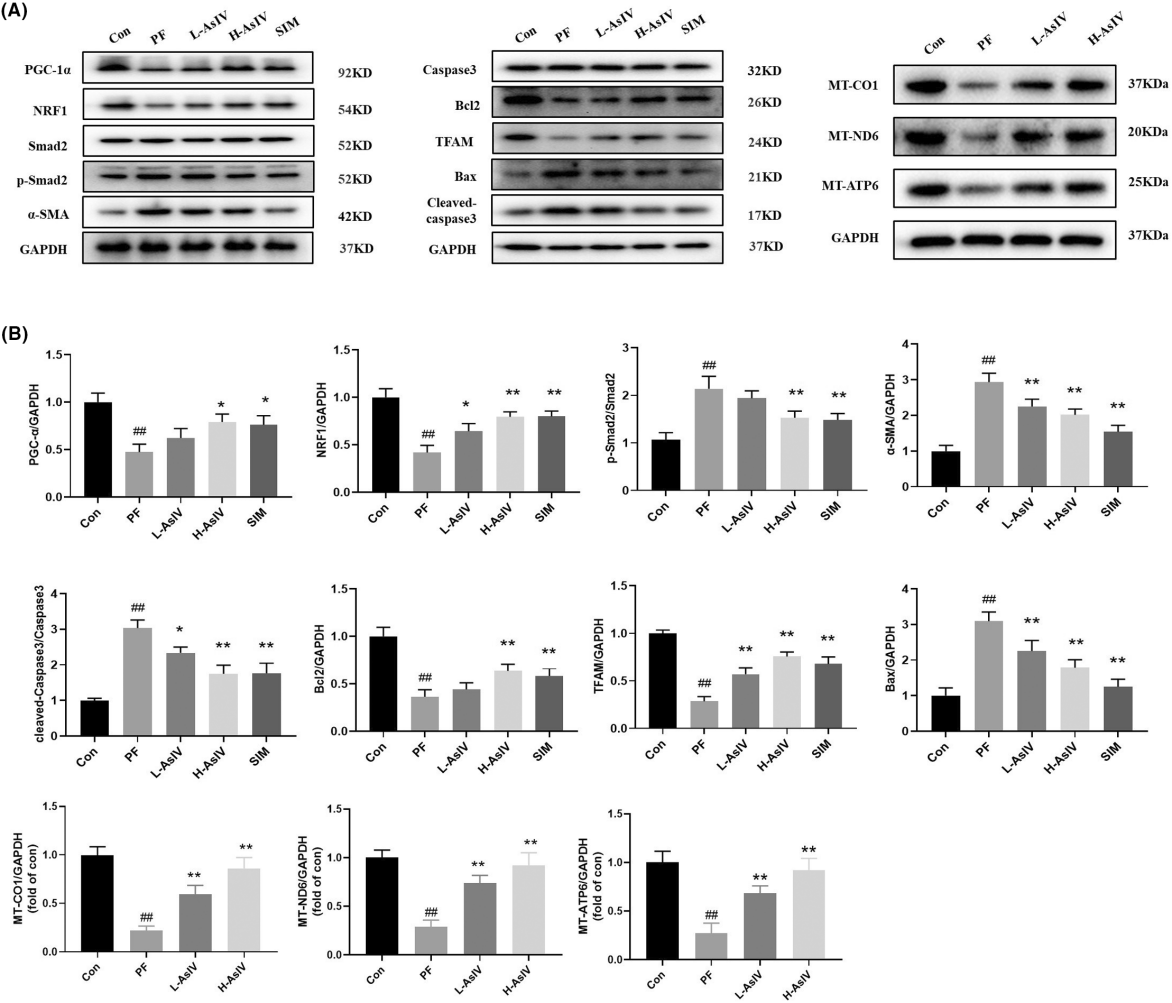
Astragaloside IV changes relative expressions of PF‐associated proteins in rats tested by western blotting. (A) Representative images of PF‐related proteins detected by western blots. (B) Relative quantification of each group of PF‐related proteins. Con: Control group; PF: PF model group; L‐AsIV: 20 mg/kg/day Astragaloside IV group; H‐AsIV: 40 mg/kg/day Astragaloside IV group; SIM: 20 mg/kg/day Simvastatin group. Results are representative of three independent experiments values are expressed as mean ± SD (*n* = 3). ^*#*^ *p* < 0.05 and ^##^ *p* < 0.01 versus Control group; * *p* < 0.05 and **p < 0.01 versus PF group.

The expressions of PGC‐1α, NRF1, Bcl2, TFAM, α‐SMA, MT‐CO1, MT‐ND6 and MT‐ATP6 were significantly increased in PF model rats treated with Astragaloside IV, while the expressions of Bax, p‐Smad2/Smad2 and Cleaved‐Caspase3/Caspase3 were significantly reduced in peritoneal tissue (Figure 2A,B). PGC‐1α, NRF1 and TFAM are mitochondrial synthesis‐associated proteins, MT‐CO1, MT‐ND6 and MT‐ATP6 were mitochondrial genes, cleaved‐caspase3 is an apoptosis‐associated protein and α‐SMA and p‐smad2/3 are fibrosis‐associated proteins. These above results suggested that Astragaloside IV can increase the synthesis of mitochondria and reduce apoptosis in the peritoneal tissue of PF rats, which corresponds to the increase of mitochondrial membrane potential and decrease of ROS level as well as TUNEL staining results in Figure 1, and also alleviates the fibrosis of peritoneal tissues, which is consistent with the results of HE and Masson's staining in Figure 1.

### 3.3 Astragaloside IV increases cell viability and reduces the apoptosis rate of cells

**FIGURE 3:**
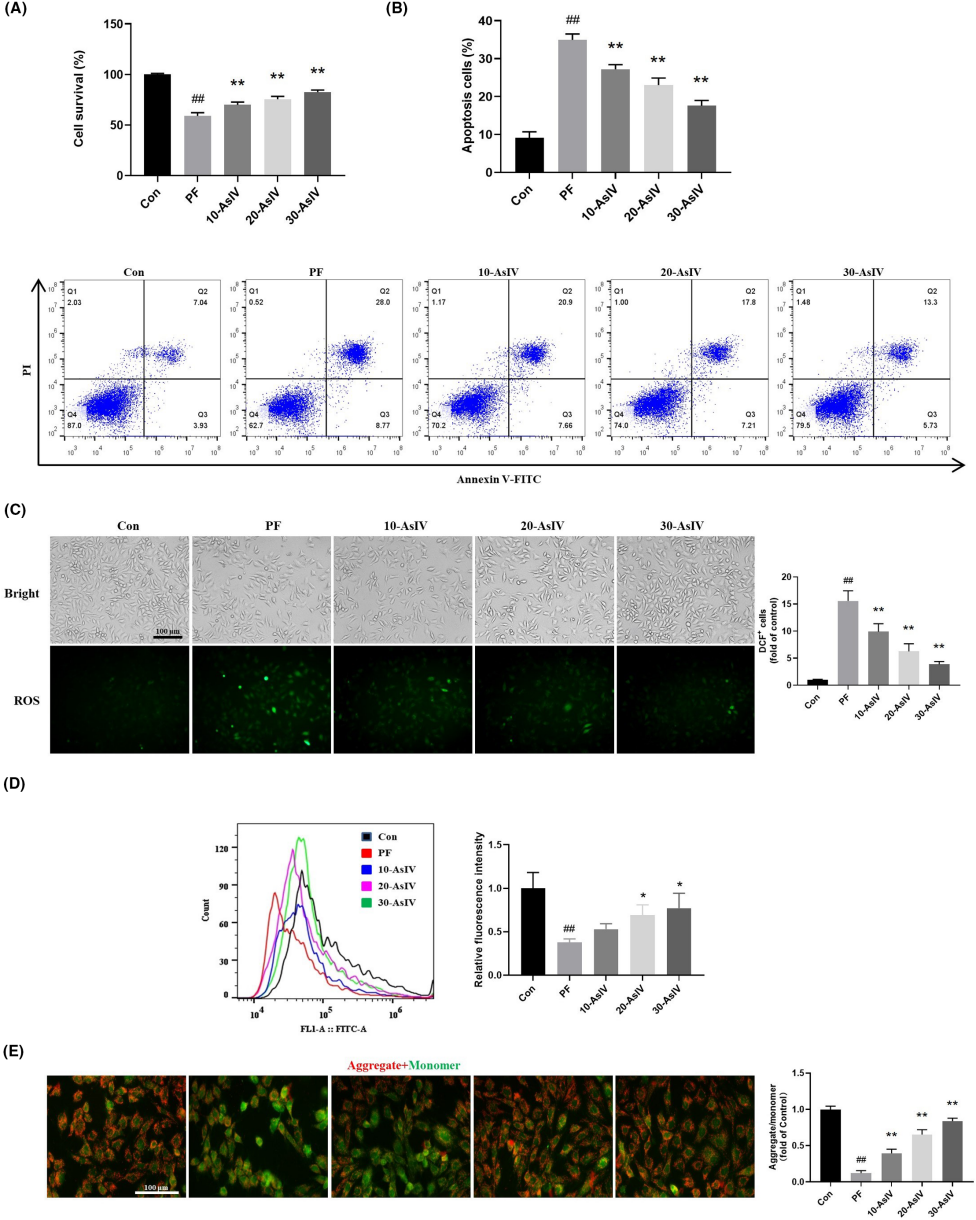
Astragaloside IV promotes the cell viability and mitochondria membrane potential and reduces the apoptosis rate of cells. (A) Astragaloside IV improves the cell viability of cells (*n* = 6). (B) The apoptotic rate of cells treated with or without Astragaloside IV (*n* = 3). (C) Relative content of DCF (ROS)‐positive cells in each group (*n* = 3). (D) The number of mitochondria in each group detected by Mito‐Tracker Green (*n* = 3). (E) Representative fluorescent images of JC‐1 staining of cells in each group (*n* = 3). Con: Control group; PF: PF model group; 10‐AsIV: 10 μg Astragaloside IV group; 20‐AsIV: 20 μg Astragaloside IV group; 30‐AsIV: 30 μg Astragaloside IV group. Results are representative of three independent experiments values are expressed as mean ± SD. ^#^ *p* < 0.05 and ^##^ *p* < 0.01 versus Control group; *p < 0.05 and **p < 0.01 versus PF group.

Figure 3A,B show that in an in vitro PF model, Astragaloside IV treatment increased cell viability and reduced the apoptosis rate of PMCs in a concentration‐dependent manner. The contents of DCF^+^ positive cells of the Astragaloside IV treatment groups were less than that of PF group (Figure 3C). As shown in Figure 3D, the relative fluorescence intensity of Mito‐Tracker Green increased with increasing concentrations of Astragaloside IV administration, indicating that Astragaloside IV promoted an increase in mitochondrial content. Red fluorescence is the aggregate JC‐1 and green fluorescence is the monomer of JC‐1. The increase in the ratio of red/green fluorescence ratio in the Astragaloside IV administered group indicated an increase in mitochondrial membrane potential (Figure 3E). The above results suggested that Astragaloside IV can improve the viability of PF model cells, enhance the depolarization of cell mitochondria and reduce apoptosis.

### 3.4 Astragaloside IV changes relative expressions of mitochondrial synthesis, apoptosis and PF‐associated proteins in cells

**FIGURE 4:**
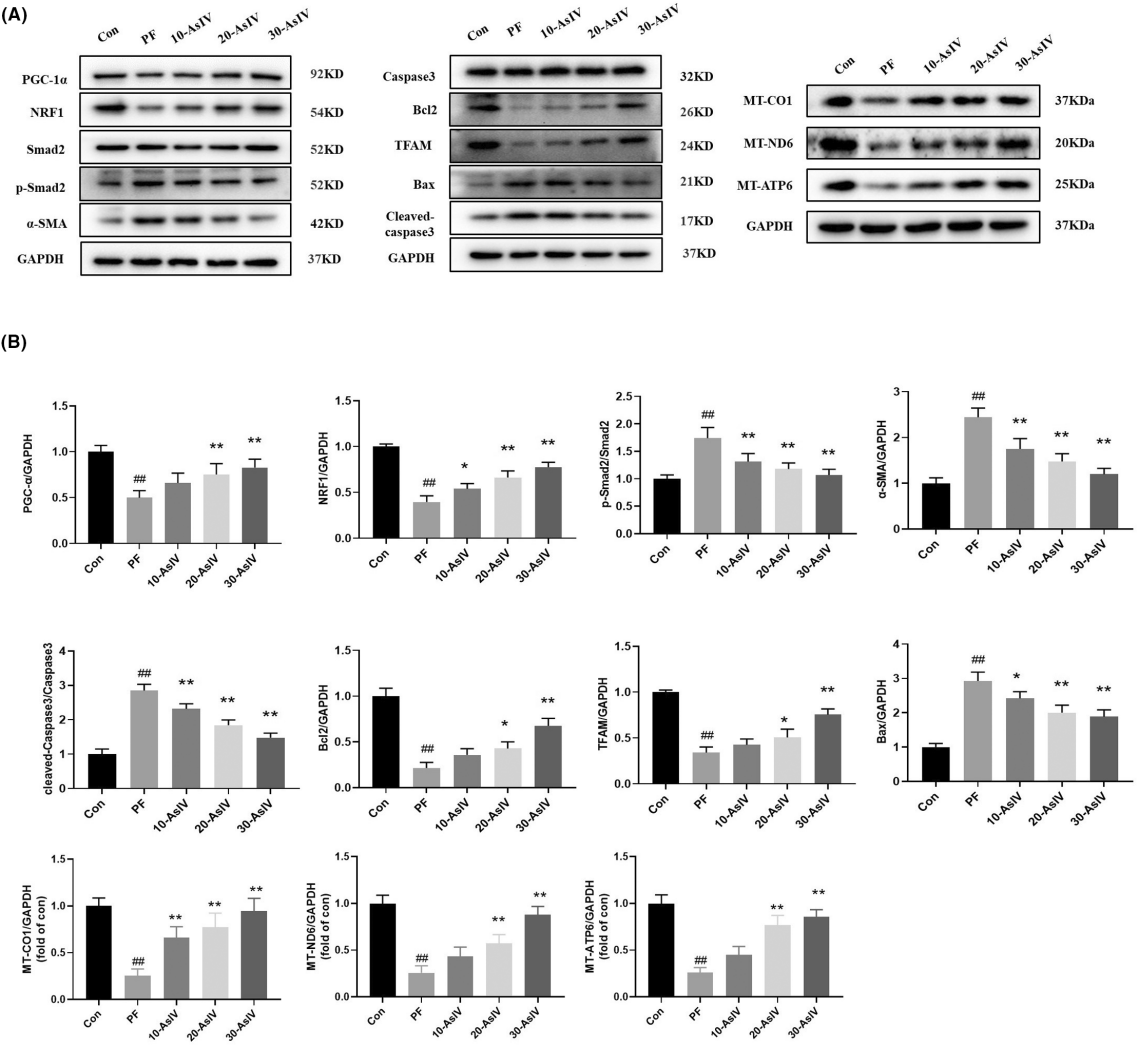
Astragaloside IV changes relative expressions of PF‐associated proteins in cells. (A) Representative images of PF‐related proteins detected by western blots. (B) Relative quantification of each group of PF‐related proteins. Con: Control group; PF: PF model group; 10‐AsIV: 10 μg Astragaloside IV group; 20‐AsIV: 20 μg Astragaloside IV group; 30‐AsIV: 30 μg Astragaloside IV group. Results are representative of three independent experiments values are expressed as mean ± SD (*n* = 3). ^#^ *p* < 0.05 and ^##^ *p* < 0.01 versus Control group; *p < 0.05 and **p < 0.01 versus PF group.

As with the in vivo assay, we also detected the expressions of these indicators by Western blot in the in vitro assay. The expressions of Bcl2, NRF1, TFAM, PGC‐1α, α‐SMA, MT‐CO1, MT‐ND6 and MT‐ATP6 were increased accompanied by increased concentration of Astragaloside IV, and Bax, Cleaved‐Caspase3/Caspase3 and p‐Smad2/Smad2 were significantly decreased in Astragaloside IV treated PMCs compared to PF group (Figure 4A,B). This proved Astragaloside IV has the same protective effect on PMCs as peritoneal tissues.

### 3.5 Astragaloside IV protects PF cells via PGC‐1α

**FIGURE 5:**
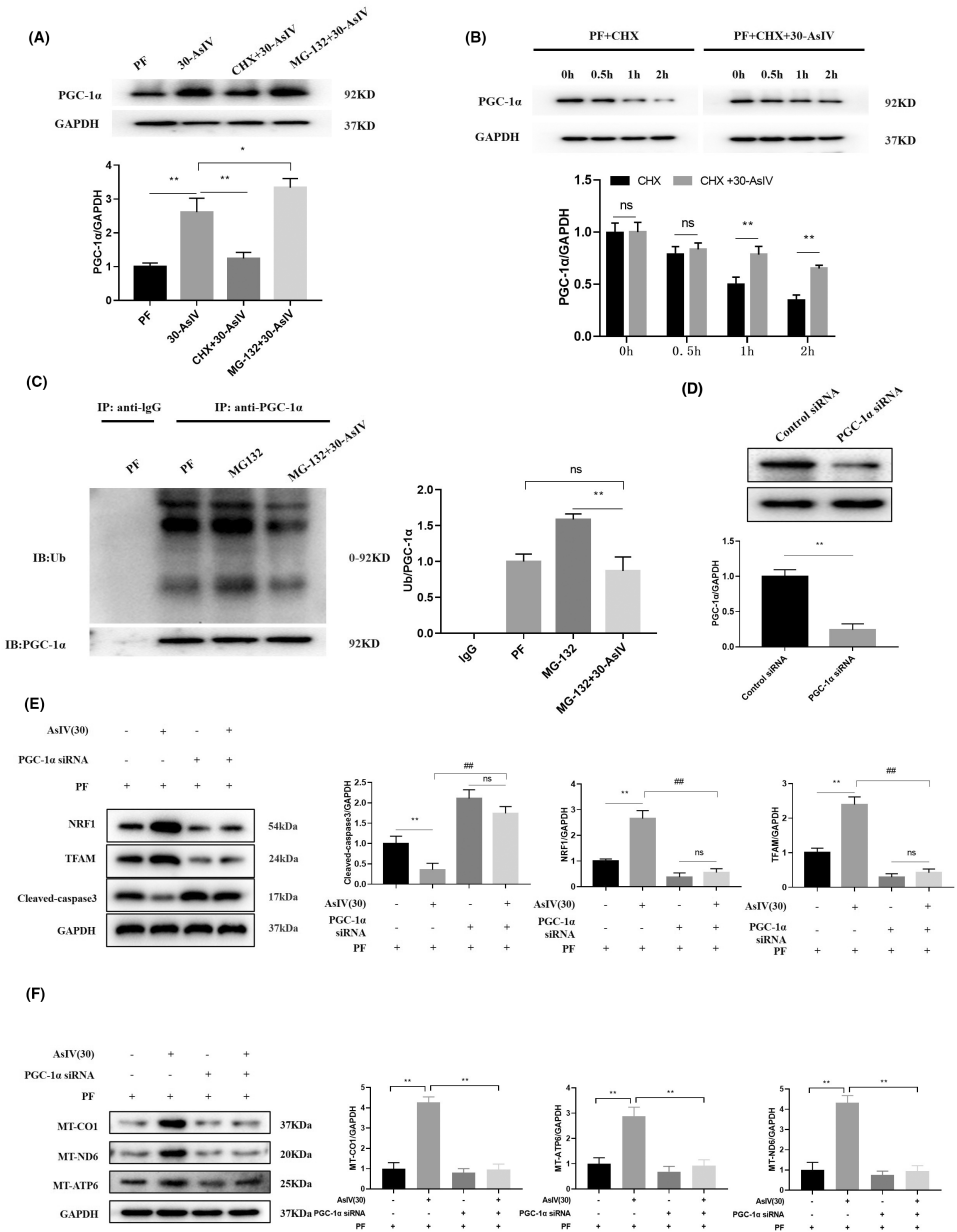
Astragaloside IV protects PF cells via PGC‐1α. (A) The increase in PGC‐1α expression caused by Astragaloside IV can be altered by CHX and MG‐132. (B) Astragaloside IV slowed the decrease in PGC‐1α expression over time. (C) Astragaloside IV stabilized PGC‐1α by reducing its ubiquitination. (D) SiRNA of PGC‐1α successfully reduced the protein expression of PGC‐1α. (E) Western blot analysis of the expressions of NRF1, TFAM and cleaved‐Caspase3 in SiRNA of PGC‐1α PMCs. (F) Western blot analysis of the expressions of MT‐CO1, MT‐ND6 and MT‐ATP6 in PGC‐1 knockdown cells PMCs. Results are representative of three independent experiments values are expressed as mean ± SD (*n* = 3). ^#^ *p* < 0.05 and ^##^ *p* < 0.01 versus Control group; *p < 0.05 and **p < 0.01 versus PF group.

MG‐132 is a selective 26S proteasome inhibitor, and Cyclohexylamine (CHX) is a chemical protein synthesis inhibitor.^16^ In Figure 5A, the results showed that CHX blocked the promotion on PGC‐1α induced by Astragaloside IV, and the expression of PGC‐1α was further promoted by MG‐132. In order to further determine the effect of Astragaloside IV on the stability of PGC‐1α protein, the half‐life of PGC‐1α protein in PMCs was calculated using western blot assay for protein expression at different time points. Indeed, Astragaloside IV treatment prolonged the half‐life of PGC‐1α compared to control (Figure 5B).

As seen in Figure 5C, MG‐132 exacerbated the ubiquitination of PGC‐1α, probably because MG‐132 inhibited the proteasome leading to the accumulation of PGC‐1α ubiquitination. After the combination of MG‐132 and Astragaloside IV, the ubiquitination of PGC‐1α was reduced, which combined with Figure 5A indicated that Astragaloside IV was used to increase the expression of PGC‐1α by reducing PGC‐1α ubiquitination. We further found that cleaved‐caspase3, which was originally reduced by Astragaloside IV, was greatly increased again in the presence of PGC‐1α was inhibited, whereas NRF1 and TFAM were significantly reduced. The expression of these proteins was not significantly affected by either the use or absence of Astragaloside IV, suggesting that the effect of Astragaloside IV on apoptosis‐ and autophagy‐related proteins was mediated through PGC‐1α action (Figure 5D,E). As shown in Figure 5F, PGC‐1α siRNA cells were treated with Astragaloside IV and the expressions of mitochondria‐related proteins were affected, and the results suggest that astragaloside IV restores mitochondrial function through PGC‐1α.

## 4 DISCUSSION

High glucose dialysis leads to excessive glycation of PMC, which results in hypoxia of peritoneal tissues, causing PF characterized by mesothelial cell detachment and extracellular matrix deposition.^17^ As peritoneal protective drugs are more directly added to the peritoneal fluid, only purified Chinese medicinal preparations can be used in clinical practice. Although some scholars have attempted to apply Chinese herbal medicines and their monomeric components such as Astragalus, ginsenosides and puerarin to combat PF, the mechanism of action and targets still need to be studied in depth.

Previous studies have revealed that the TGF‐β/Smads pathway is important in the fibrosis process,^18^, ^19^ and the mechanism of anti‐fibrosis of Astragalus is mainly manifested in the inhibition of the TGF‐β1/Smads pathway.^13^ Our previous studies also showed that Astragalus can downregulate p‐Smad2/3, enhance inhibitory Smad7 and block epithelial‐mesenchymal transition in rat peritoneal cells.^14^, ^15^ The effectiveness of Astragalus against PF was confirmed and the mechanism of Astragalus was also explored, mostly in agreement with the literature. However, a contradiction has been found, the Astragalus did not promote Smad7 as well as smad2/3.^15^ Therefore, we speculate that Astragalus may inhibit PF through other related pathways besides TGF‐β1/Smads.

As apoptosis is a direct factor in PF and mitochondrial dysfunction is closely related to apoptosis, promoting mitochondrial biosynthesis and functional protection in PMCs is expected to provide a new strategy for delaying or even reversing PF. There are two ways to avoid apoptosis due to mitochondrial dysfunction: one is to increase the number of respiratory chains to disperse electrons, reducing the probability of electron retention in the mitochondrial complex and decreasing the accumulation of ROS^20^; the another is to improve the activity of regulatory factors within the mitochondria to maintain intracellular physiological homeostasis and regulate intracellular ROS levels.^21^ Promoting mitochondrial biosynthesis is therefore particularly important to reduce oxidative damage and maintain cellular function. PGC‐1α is a major regulator of mitochondrial synthesis and oxidative metabolism, thus PGC‐1α may be a new target for PF inhibition.^5^ We speculate that Astragaloside IV likely affects PF via a mitochondrial biosynthesis‐ROS‐apoptosis‐related pathway.

Previous studies have found that the addition of Astragalus injection to the PD solution increased the amount of dialysis ultrafiltration and improved peritoneal clearance of solutes.^13^ However, these reports have not been further investigated systematically, and few articles have reported the effect of Astragalus on mitochondrial biosynthesis‐ROS‐apoptosis correlation. In this study, a PF rat model was constructed by injecting 4.25% glucose dialysis solution, and the abdominal tissue of the model rats exhibited thickened peritoneal fibres, which was consistent with the results of previous study, indicating that the PF model was successfully constructed.^15^ In vivo experiments suggested that Astragaloside IV inhibited ROS accumulation and apoptotic rate, increased mitochondrial membrane potential. Through the detection of protein expression, it was found that Astragaloside IV also increased the expression of PGC‐1α and mitochondrial synthesis proteins, and decreased the expression of fibrosis‐related and apoptosis‐related proteins.

To further verify whether this is through the mitochondrial biosynthesis‐ROS‐apoptosis pathway, we constructed a cellular PF model of PMCs and obtained results similar to those of in vivo experiments. Astragaloside IV was found to increase the expression of PGC‐1α by reducing the ubiquitination of PGC‐1α. And when PGC‐1α was inhibited, the effect of Astragaloside IV on mitochondrial synthesis‐related and apoptosis‐related proteins was blocked, and thus the protective effect of PMCs was prevented. Our findings suggested that Astragaloside IV has a protective effect and therapeutic potential in PD‐induced fibrosis. Combined with these results, we hypothesized that the molecular mechanism of Astragaloside IV against PF is via regulating PGC‐1α, promoting mitochondrial biosynthesis, regulating excess ROS and blocking peritoneal mesothelial cell apoptosis, thereby preventing and treating PF.

However, there are some limitations in this study. First, we did not study the specific mechanism of Astragaloside IV on PF, but only explored the possibility of astragaloside IV acting through the mitochondrial biosynthesis‐ROs‐apoptosis pathway or other pathways. Second, there is a lack of positive control group in cell experiments. After our preliminary exploration this time, drugs that promote mitochondrial biosynthesis can be considered as positive control in the next step. In addition, other cell death modes besides apoptosis also promote the progression of PF. For example, PF induced by high glucose solution in peritoneal mesenchymal cells of mice is accompanied by increased expression of LC3 II/I (biomarker of autophagy)^22^; the inhibitor of ferroptosis can inhibit the progression of PF in rats.^23^ These pathways also interfere with PF, and further studies should be conducted to explore whether Astragaloside IV inhibits PF through these pathways.

## 5 CONCLUSION

In conclusion, we demonstrated that Astragaloside IV can effectively improve fibrosis in rats and PMCs PF model. Selective blockade of the PGC‐1α/ROS/apoptosis signalling pathway may be a mechanism by which Astragaloside IV ameliorates PF. To our knowledge, this is the first study to show that Astragaloside IV can inhibit fibrosis via the PGC‐1α/ROS/apoptosis pathway.

## AUTHOR CONTRIBUTIONS

**Mingxia Xie:** Data curation (equal); formal analysis (equal); funding acquisition (equal); methodology (equal); project administration (equal); validation (equal); writing – original draft (equal). **Bohou Xia:** Data curation (equal); methodology (equal); validation (equal). **Lan Xiao:** Data curation (equal); methodology (equal); validation (equal). **Dun Yang:** Data curation (equal); methodology (equal); validation (equal). **Zhenghong Li:** Data curation (equal); methodology (equal); validation (equal). **Hanqing Wang:** Formal analysis (equal); software (equal); validation (equal). **Xiaoye Wang:** Software (equal); validation (equal). **Xi Zhang:** Conceptualization (equal); validation (equal); writing – original draft (equal); writing – review and editing (equal). **Qinghua Peng:** Conceptualization (lead); funding acquisition (equal); validation (equal); writing – review and editing (equal).

## FUNDING INFORMATION

This work was supported by the 2022 “Disciplinary Reveal System” project of Hunan University of Chinese Medicine (22JBZ024); Key Projects of Hunan Provincial Department of Education (22A0259); China Postdoctoral Science Foundation (2021M690990); The 2021 “Academician Liu Liang Workstation” Guidance Project (21YS002); Open Fund Project of State Key Laboratory Cultivation Base Jointly Constructed by Ministry and Province (2022FTKFJJ09); Hunan Province Clinical Medical Technology Innovation Guidance Project (2021SK50809); Key Project of the First‐Class Subject Open Fund of the Education Department of Hunan Province (2020ZXYJH12).

## CONFLICT OF INTEREST STATEMENT

The authors declare no conflict of interest.

## Data Availability

Data available on request from the authors.
